# Effects of an interprofessional deprescribing intervention in Swiss nursing homes: the Individual Deprescribing Intervention (IDeI) randomised controlled trial

**DOI:** 10.1186/s12877-021-02465-7

**Published:** 2021-11-19

**Authors:** Damien Cateau, Pierluigi Ballabeni, Anne Niquille

**Affiliations:** 1 Community Pharmacy, Centre for Primary Care and Public Health (Unisanté), University of Lausanne, Rue du Bugnon 44, CH-1011 Lausanne, Switzerland; 2 School of Pharmaceutical Sciences, University of Geneva, Geneva, Switzerland; 3 Institute of Pharmaceutical Sciences of Western Switzerland, University of Geneva, University of Lausanne, Geneva, Switzerland; 4 Division of Biostatistics, Centre for Primary Care and Public Health (Unisanté), University of Lausanne, Lausanne, Switzerland

**Keywords:** Deprescribing, Nursing home, Collaboration, Potentially inappropriate medications, Medication review

## Abstract

**Background:**

Deprescribing polypharmacy and potentially inappropriate medications (PIMs) has been shown to be beneficial to nursing home (NH) residents' health. Medication reviews are the most widely studied deprescribing intervention; in a previous trial, we showed that another intervention, a deprescribing-focused interprofessional quality circle, can reduce the use of inappropriate medications at the NH level.

However, this intervention cannot account for the variety of the residents’ clinical situations. Therefore, we trialled a subsequent intervention in NH that enacted the quality circle intervention in the previous year.

**Methods:**

In 7 NHs, the most heavily medicated residents were recruited and randomised to receive usual care or the intervention. The intervention was a pharmacist-led, deprescribing-focused medication review, followed by the creation of an individualised treatment modification plan in collaboration with nurses and physicians.

Intervention’s effects were assessed after four months on the number and dose of PIMs used, quality of life, and safety outcomes (mortality, hospitalisations, falls, and use of physical restraints). Data were analysed using Poisson multivariate regression models.

**Results:**

Sixty-two NH residents participated, falling short of the expected 100 participants; 4 died before initial data collection. Participants used a very high number of drugs (median 15, inter-quartile range [12-19]) and PIMs (median 5, IQR [3-7]) at baseline. The intervention did not reduce the number of PIMs prescribed to the participants; however, it significantly decreased their dose (incidence rate ratio 0.763, CI_95_ [0.594; 0.979]), in particular for chronic drugs (IRR 0.716, CI_95_ [0.546; 0.938]). No adverse effects were seen on mortality, hospitalisations, falls, and restraints use, but, in the intervention group, three participants experienced adverse events that required the reintroduction of withdrawn treatments, and a decrease in quality of life is possible.

**Conclusions:**

As it did not reach its recruitment target, this trial should be seen as exploratory. Results indicate that, following a NH-level deprescribing intervention, a resident-level intervention can further reduce some aspects of PIMs use. Great attention must be paid to residents’ well-being when further developing such deprescribing interventions, as a possible reduction in quality of life was found in the intervention group, and some participants suffered adverse events following deprescribing.

**Trial registration:**

ClinicalTrials.gov (NCT03688542, https://clinicaltrials.gov/ct2/show/NCT03688542), registered on 31.08.2018.

**Supplementary Information:**

The online version contains supplementary material available at 10.1186/s12877-021-02465-7.

## Introduction

### Background

Polypharmacy (the concurrent use of multiple drugs) and the use of potentially inappropriate medications (PIM) are widely seen as a threat to health, particularly in older patients, which are more sensitive to their detrimental effects [1, 2]. Deprescribing, the structured withdrawal of non-beneficial drugs, has been conceptualised as a solution to both problems [3]. In the last decade, evidence of its beneficial effects has accumulated: recent meta-analyses have shown that deprescribing interventions can reduce mortality, both in the general geriatric population and in nursing home (NH) residents, [4, 5] and falls in NH residents [5].

Not all deprescribing interventions, however, have been equally successful at improving outcomes: both meta-analysis found that medication reviews, a systematic, patient-centred process, [6] were more likely to produce positive outcomes than other interventions [4, 5].

### Local context and research project

In two cantons of Western Switzerland, NHs are required to implement an integrated pharmacist service (IPS), which structures the collaboration of nurses, physicians and pharmacists in the home. A major part of this IPS are regular meetings organised and facilitated by the pharmacist. Based on the quality circle methodology, these meetings gather nurses, physicians and pharmacists one to four times a year to discuss the current use of drug classes (e.g. antihypertensives, pain management, diabetes) in the NH [7]. Discussions during these quality circles are based on evidence and guidelines gathered from the literature, and conducted in light of drug use statistics prepared by the pharmacist, with the goal of improving drug choices and drug use in the NH, and reducing drug costs [8, 9].

These existing collaborations have been used to trial a deprescribing intervention targeting widely used PIMs through the implementation of deprescribing consensus crafted during quality-circle sessions. The protocol and results of this first trial, called Quality Circle Deprescribing Module (QC-DeMo), have been published [10, 11]. QC-DeMo succeeded in reducing the use of some classes of PIMs, such as proton-pump inhibitors (PPI), but did not reduce overall PIM usage. Thus, as anticipated, many NH residents still received PIMs after this first intervention, as the diversity of their clinical conditions could not be addressed by an intervention targeted at the NH level. Therefore, one year after the QC-DeMo intervention, a second trial was launched, investigating an Individual Deprescribing Intervention (IDeI), targeting the residents prescribed the most medications in the NHs that enacted the QC-DeMo intervention. We hypothesised that this second intervention would result in a reduction of the number of PIMs prescribed to these residents.

## Methods

### Setting

The IDeI trial took place in the NHs allocated to the intervention group of the QC-DeMo trial and that volunteered for this second trial [10]. Agreement of all implicated nurses, physicians and pharmacists were required for the NH to take part in the trial.

### Population and recruitment

All residents living in a volunteer NH for at least four months, aged 65 years or more, and prescribed five or more medications were eligible for the IDeI study. No formal exclusion criteria were enacted, although the NH staff could choose not to offer participation to a specific resident if discussing the possibility of taking part in the trial would cause undue distress to them or their relatives.

Recruitment was carried out by the NH staff: pharmacists ranked all NH residents from the largest to the smallest number of prescribed medications; nurses then offered participation to the residents following this ranking, except to those previously excluded as described above, until 20% of the population of the NH had been recruited. In case of cognitive impairment, participation in the trial was discussed with the resident's representatives. This recruitment method was chosen to ensure that residents receiving the most drugs, an easy to assess proxy for PIMs use, [12, 13] and thus the most likely to benefit from the intervention, were recruited.

### Randomisation and blinding

Participants were randomised between the intervention and control groups at the time of inclusion, in a 1:1 ratio at the level of the NH. For each volunteer NH, a randomisation list of length equal to 20% of the number of beds of the NH was generated by the investigators, using the tool provided at https://www.randomisation.com. These lists were created using randomly permutated blocks of size 2, to ensure equilibrium between groups even in case of incomplete recruitment in the NH, and then uploaded into REDCap (Research Electronic Data Capture) [14, 15]; randomisation happened upon completion of the inclusion questionnaire hosted on REDCap.

Given the nature of the intervention, NH staff (pharmacists, physicians and nurses) could not be blinded to the allocation. As the data collected differ between participants in the intervention and control group (see Data collection), investigators could not be blinded either. Thus, only the statistician was blinded; unblinding occurred only after analysis completion.

To limit the bias introduced by the unblinded nature of the intervention, the main outcome was assessed using a validated tool (see Outcomes).

### Intervention

Prior to the start of the trial, the pharmacists took part in a 3-day postgraduate education session on the methodology of performing medication reviews, as this is not part of the pharmacy curriculum in Switzerland.

The intervention consisted of a deprescribing-focused medication review, performed by the pharmacists, followed by the creation of a treatment modification plan in collaboration with nurses and physicians. Once agreed upon by the professionals, the plan was submitted to the participating resident, or her/his representative, before implementation.

Pharmacists were provided with standardised forms for performing the medication review and documenting the treatment modification plan; the same form was used to collect subsequent modifications to the participants’ medication (dose change, re-start of a stopped drug, etc.) Adherence to the intervention was verified by the regular collection of these forms by the investigators.

### Comparator

Participants allocated to the control group were cared for as usual; in particular, their drug regimen could be adapted freely by the NH staff and physicians for the duration of the study.

### Outcomes

The main outcome was the number of PIMs used at baseline and four months after intervention; PIM status was assessed using the French translation of the screening tool of older people’s prescriptions (STOPP), version 2 [16].

Secondary outcomes were the number of chronic (not in reserve or short-term treatment) drugs, the number of inappropriate defined daily doses (DDD) per day, the number of chronic DDD per day, the number of common complaints frequently related to drug use, and health-related quality of life (QoL), measured with EuroQol-5 Dimension-5 Levels (EQ-5D-5L), all assessed at baseline and four months after intervention [17]. For the latter outcome, both the results of the visual analogue scale and the index value were used; the index was valued using the French value set [18]. An exploratory analysis of the evolution of potentially inappropriate chronic DDDs was performed.

Safety outcomes were the mortality rate, the number of participants hospitalised, the number of days spent in hospital, the number of participants having experienced at least one fall, the number of falls, and the number of days spent with physical restraints.

The impact of the intervention on NHs staff, measured using the NeuroPsychiatric Inventory-Nursing Home, and drug costs will be reported in a future paper examining the implementation of the intervention. Aspects such as the reasons for refusal of pharmacists’ propositions, and who refused them (physician or participant) will be addressed in this paper as well.

### Data collection

Data were collected by the NH pharmacists (treatment plans, STOPP/START analysis, intervention-specific data for implementation evaluation) and nurses (quality of life, common complaints, deaths, hospitalisations, falls and physical restraints use); data were collected on paper forms, scanned by the pharmacist, and sent to the investigators via REDCap, who then transcribed them using electronic case report forms.

Baseline data collection occurred, for both groups, at the implementation of the first deprescribing measures in the intervention group, and follow-up data collection occurred four months later. For participants hospitalised at the time of follow-up, their last treatment plan in the NH was considered for follow-up; in case of death, the treatment plan ten days before death was considered, to avoid taking into account the treatment modifications happening at the end of life independently of the intervention. In both cases, no QoL and common complaints questionnaires were collected.

### Sample size

As no data on the use of PIMs by individual residents in the NHs of Vaud and Fribourg were available prior to the trial, no sample size calculation was performed. Ten NHs were expected to take part in the study; with an average of 50 beds per NH and a 20% recruitment target, the anticipated number of participants was 100.

### Statistical analysis

The comparison of the primary outcome (number of PIMS four months after intervention) between groups was performed by means of Poisson regression under adjustment for baseline values of the outcome. All other outcomes were analysed in the same way, using either ordinary least square (OLS) regression or Poisson regression according to outcome and residual distributions. Binary safety outcomes were analysed by means of Fisher’s exact tests, quantitative safety outcomes by means of Wilcoxon-Mann-Whitney tests. The analyses were performed with Stata version 16.0 (StataCorp LLC, College Station, TX, USA).

### Ethical considerations and reporting

The IDeI trial was authorised by the *Commission cantonale d’éthique de la recherche sur l’être humain* for the Canton of Vaud, the relevant ethics committee (decision 2018 − 01279), and prospectively registered on ClinicalTrials.org (NCT03655405) and on the Swiss national registry of clinical trials (SNCTP000002975), as required by Swiss law.

The CONSORT extensions for the reporting of pragmatic trials and the reporting of non-pharmacologic treatments trials were followed for the preparation of this article [19, 20].

## Results

The IDeI trial took place in seven NHs (4 from Vaud, 3 from Fribourg), with a diversity of missions and sizes (see Table 1); the 11 eligible NHs that elected not to take part in the study cited the lack of time of their staff (9 NHs) or the complexity of the study (2 NHs) as a reason.

**Table 1:** characteristics of NHs and participants

**Characteristics of NHs**			
Canton			
Vaud	4		
Fribourg	3		
NH mission			
Geriatric	3		
Psycho-geriatric	3		
Mixed*	1		
Number of beds ^a^	65 (49.5–83)		
Attending physicians ^a^	1 (1–2)		
**Baseline characteristics of participants**			
	**Intervention** ***n*** **= 31**^**†**^		**Control** ***n* = 27**^**‡**^
Female gender ^b^	16 (52%)		20 (74%)
Age ^a^	87 (80–91)		84 (78–88)
Duration of stay in years ^c^	3.1 (2.3)		3.6 (3.6)
Cognitive impairment ^b,§^	10 (32%)		9 (33%)
Number of drugs prescribed ^a^	15 (12-19)		15 (13-18)
of which: chronic drugs ^a^	12 (10-14)		11 (8-12)
of which: PIMs ^a^	5 (4-7)		6 (3-7)
Number of chronic DDDs ^a^	9.6 (3.5)		9.3 (4.8)
Number of PIM DDDs ^a^	3.7 (2.9)		3.6 (2.1)
Quality of life			
EQ-5D-5L analogue scale ^a^	56.5 (24.8)		61.4 (20.5)
EQ-5D-5L index ^a^	0.46 (0.34)		0.50 (0.33)

*NH* nursing home; *PIM* potentially inappropriate medication; *DDD* defined daily dose; *EQ-5D-5L* EuroQol-5 Dimensions-5 Levels; *IQR* Inter-Quartile Range; *SD* Standard Deviation; *: both geriatric and psycho-geriatric population; §: unable to give consent, consent given by representative; †: 1 participant excluded, died before initial data collection; ‡: 3 participants excluded: 2 died before initial data collection, no data was collected on one

a: median [IQR]; b: n (%); c: mean (SD)

**Fig. 1:**
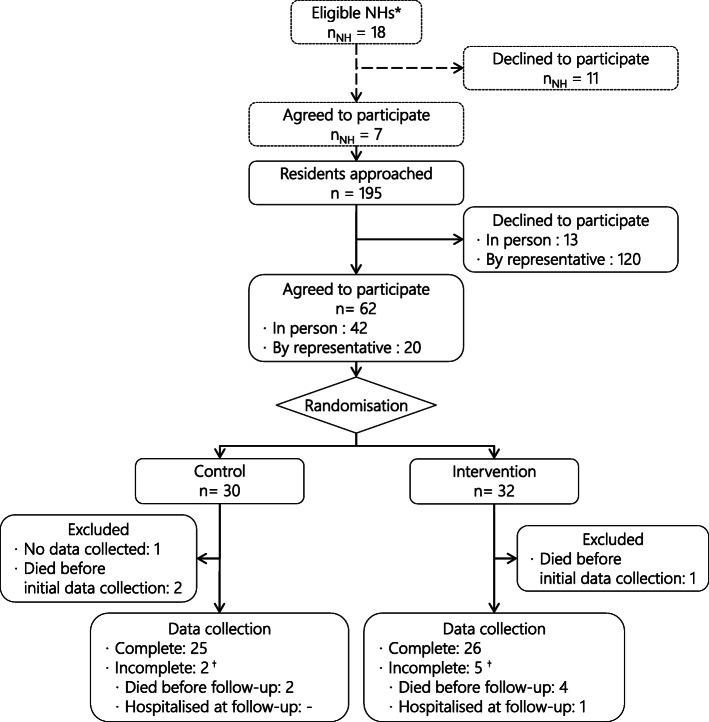
Flow-chart for the IDeI trial. NH: nursing home; *: NHs allocated to the intervention group in the QC-DeMo trial; †: only the treatment-related outcomes could be assessed

One hundred and ninety-five residents of these NHs were approached by the nursing staff to take part in the study (recruitment period: October 2018 to March 2019), and 62 agreed (42 themselves, 20 via a representative). Figure 1 details the flow of the study and Table 1 the baseline characteristics of participants; initial data collection occurred, on average, 50 days after inclusion in the intervention group, and 37 days in the control group. Both ages and gender balance of the participants were consistent with the population of their NHs. Participants in the two groups rated their baseline quality of life as medium according to EQ-5D-5L, both with the analogue scale and after valuation of their answers to the questionnaire. They used a high number of drugs, which is easily explained by the recruitment strategy, and, like hypothesised, an important proportion of those were PIMs, with a median number of five, respectively six, identified using STOPP v2 in the intervention and control group. Dosing tended to be, on average, lower than for younger adults, as the number of DDDs was inferior to the number of drugs, both for all drugs and PIMs.

At baseline, all participants except one were prescribed at least one drug meeting one STOPP criterion (98% prevalence); the most prevalent STOPP criteria were those related to the use of anticholinergic medication, with over 90% of participants using a combination of them, to the long-term use of benzodiazepines (72% of participants), and to the use of neuroleptics (36%). START criteria were less common, with only 31 participants meeting one at baseline (53% prevalence); the most prevalent were those concerning the use of vitamin D or bone anti-resorptive therapy. Supplementary Table 1 details the STOPP and START criteria present at baseline and their change at follow-up.

### Treatment changes resulting from the intervention

One hundred and sixty-nine treatment modifications were proposed by the pharmacists to the participants’ physicians and the nursing staff; 82 (49%) were accepted and implemented, and 67 of these were sustained at follow-up. The most frequent propositions concerned pain medications (20 propositions, 10 accepted, 9 sustained), benzodiazepines (16 propositions, 6 accepted, 3 sustained), PPIs (13 propositions, 6 accepted, 5 sustained), and drugs reducing blood pressure (11 propositions, 3 accepted, 3 sustained). The most common propositions were stopping a drug (55 propositions, 43 accepted, 41 sustained), reducing a dose (22 propositions, 10 accepted, 9 sustained) and replacing a drug with another, more appropriate one (18 propositions, 4 accepted, 4 sustained).

### Impact of the intervention

Prevalence of PIMs (55 participants, 95% prevalence) and START criteria (35 participants, 56% prevalence) remained high in both groups at follow-up; the difference between groups was not tested, as these were not pre-specified outcomes.

**Fig. 2:**
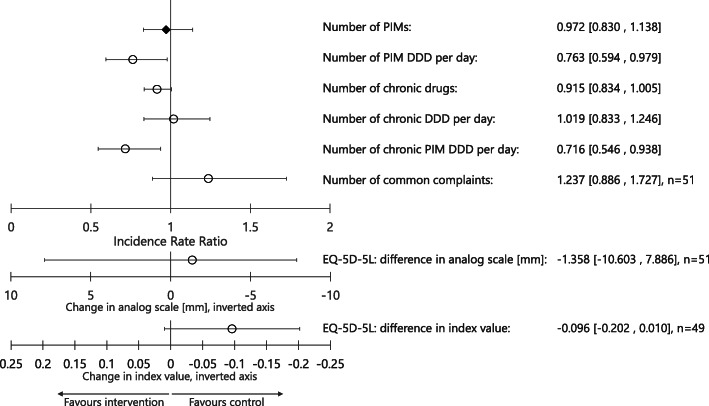
effect of the intervention on the use of PIMs and chronic drugs; n = 58 unless otherwise specified; all analysis performed with Poisson regression (incidence rate ratio reported), except for EQ-5D-5L (linear regression). PIM: potentially inappropriate medication; DDD: defined daily dose; EQ-5D-5L: EuroQol-5 Dimensions-5 Levels

The intervention did not have a statistically significant impact on the number of PIMs (incidence rate ratio (IRR) 0.972, 95% confidence interval [0.83, 1.138]) or of chronic drugs used at follow-up (IRR 0.915, 95% CI [0.834, 1.005]) (see Fig. 2). A strong effect was seen on the amount of PIMs used, with the number of PIM DDDs significantly reduced in the intervention group (IRR 0.763, 95% CI [0.594, 0.979]); the effect was more noticeable when looking only at chronic drugs, with a 28 % reduction (IRR 0.716, 95% CI [0.546, 0.938]) in the number of long-term PIM DDDs.

In both groups, a trend towards a reduction in the number of common complaints and an increase in QoL between baseline and follow-up was present; this trend was less marked in the intervention group, particularly for the QoL index valuation, leading to the difference with the control group nearly reaching statistical significance (see Fig. 2).

### Safety of the intervention

More participants died in the intervention group (4) than in the control group (2); however, this difference did not reach statistical significance, and no death in the intervention group was imputed to the consequences of a treatment change resulting from the intervention: one died from advanced prostate cancer, and the other three following several weeks of general physical decline.

The intervention did not affect the other safety outcomes (falls, hospitalisations, and use of physical restraints; see Table 2), although, due to the low number of observations, no formal test could be performed for length of hospitalisation.

**Table 2:** Impact of the intervention on safety outcomes

	Intervention *n* = 31 ^†^	Control *n* = 27 ^‡^	p for difference between groups
Number of deaths (n, % in group)	4 (13%)	2 (7%)	0.675 ^a^
Hospitalisations			
Participants hospitalised (n, % in group)	3 (10%)	1 (4%)	0.615 ^a^
Days spent in hospital (mean, SD)	3.6 (15.8)	0.6 (2.9)	-^c^
Falls			
Number of falls per participant (mean, SD)	0.45 (0.89)	0.48 (0.80)	0.738 ^b^
Number of participants who fell (n, % in group)	9 (30%)	9 (33%)	0.781 ^b^
Number of falls in participants that fell at least once (mean, SD)	1.6 (1.0)	1.4 (0.7)	0.958 ^b^
Number of days spent with physical restraints (mean, SD)	15.6 (39.1)	15.1 (39.1)	0.911 ^b^

†: 1 participant excluded, died before initial data collection; ‡: 3 participants excluded: 2 died before initial data collection, no data was collected on one

a: Fisher’s exact test (too few events to perform logistic regression); b: Wilcoxon-Mann-Whitney ranksum test; c: too few data for a meaningful statistical test

Three participants in the intervention group experienced adverse events resulting from a treatment modification: one experienced hematemesis following the reduction of esomeprazole dosing from 40 to 20 mg (the dose was increased again following the event); one participant suffered a gout flare two weeks after their febuxostat treatment was reduced from 80 to 40 mg a day (the flare was treated with prednisone and febuxostat 80 mg subsequently restarted); the third participant suffered from leg oedemas after discontinuation of their torasemide treatment (torasemide was restarted at the previous dose). All participants recovered fully before the end of the study.

## Discussion

This trial shows that an interprofessional deprescribing intervention can reduce the doses of PIMs used by NH residents, particularly for long-term medications, without increasing mortality, falls or hospitalisations. However, the number of PIMs prescribed was not affected by the intervention, and three participants in the intervention group experienced adverse consequences from deprescribing.

### Characteristics of the population

Both the number of drugs and the prevalence and number of PIMs were higher in our study than in other involving similar participants and using similar PIM assessments. Frakenthal and coll., for example, in a study of 359 residents of a chronic care facility, [21] had only 15% of participants receiving 13 or more drugs, whereas our median number of drugs was 15 at baseline; Garcia-Collarte and coll. found that only 29 % of the participants in their study had 11 or more drugs prescribed [22]. Regarding the prevalence of PIMs, other studies that used the STOPP criteria to assess appropriateness reported values ranging from 39 to 89%, [23] while we found that all but one participants in our study received at least one PIM at baseline (98% prevalence). For the number of PIMs per participant, Garcia-Gollarte and coll. reported a mean of 1.1 per participant at baseline, [22] compared to a median of 5, respectively 6, in our intervention and control groups.

Multiple factors can explain these findings; first, our recruitment strategy specifically targeted NH residents receiving a high number of medications, as this parameter was found to be a good predictor of PIMs prescription [12, 24]. Second, we used the second version of STOPP/START, published in 2015, [25] whereas most published studies use the older version 1 [26]. While these two versions have many similarities, they also differ in substantial ways: version 2 includes more STOPP criteria (85) than the previous one (65), which leads to the identification of more potentially inappropriate medications in the same population [27]. A significant addition to the version 2 was criteria N1, relative to the simultaneous use of multiple anticholinergic drugs; this criteria turned out to be the main driver of inappropriateness in our trial, with over 90% of participants meeting it at baseline. Multiple large trials using the STOPP/START v2 to assess appropriateness in populations similar to ours are currently underway, [28, 29] and it will be interesting to see if this new version of the criteria has the same impact on the prevalence of PIMs as seen here. Finally, while our population is comparable in terms of age to other studies conducted in NHs, it is possible that it differs in terms of health status and issues. Switzerland has indeed a very active policy of enabling older people to live at home for the longest time possible with the help of home care services, [30] which could lead to the population of its nursing homes being frailer than in other countries without such a policy, in turn increasing the number of drugs prescribed. However, very few data that would enable such a comparison are available, and the few that are do not indicate that Swiss NH residents are in worse health than their peers in other countries [31].

### Impact of the intervention

#### On medications

The intervention in this study did not significantly reduce the number of either PIMs or overall drugs used by participants, in contrast with trials of similar interventions where successful reduction of the number or prevalence of PIMs occurred, [22] where the number of total drugs were reduced by the intervention, [32] or where a greater proportion of participants in the intervention groups successfully stopped at least one PIM [33, 34]. The small sample size of our study certainly contributed to this difference: most other studies included a significantly larger number of participants, although Potter and coll. demonstrated a significant reduction of the total number of drugs in a trial where only 100 NH residents took part [32]. Another factor was our recruitment strategy: we hypothesised that the residents receiving the most drugs would benefit the most from the intervention, but it could also be the case that their treatment had already been optimised as much as possible, which would prevent any significant improvement from being made.

Finally, this study took place in NHs in which, a year earlier, another deprescribing intervention was conducted (QC-DeMo); this first intervention was meant to reduce the use of some widely-used PIMs, such as the long-term use of PPIs, in the whole NH, and did not focus on individual residents. This NH-wide intervention could have sufficiently reduced the use of some PIMs to render the medication review performed in this study unnecessary. An example of this possible effect is seen on the use of PPIs: we found a very low rate of long-term PPI use (9% at baseline across both groups, see Supplementary Table 1) compared to other studies, [35, 36] and all NHs where the IDeI trial took place had enacted a consensus to reduce the use of PPIs as a result of their participation in the QC-DeMo trial [11].

#### On QoL and common complaints

The EQ-5D-5L questionnaire was chosen for QoL measurement for its simplicity of use and the possibility to administer it by proxy. Despite not being specifically designed for use with older people with possible cognitive impairment, it has been favourably compared with more specialised tools such as QoL-AD and DEMQoL [37, 38]. In line with the conclusions of a recent meta-analysis, [39] we expected to see no effect of our intervention on participants’ QoL; while the difference seen between the two groups did not reach statistical significance, there was a strong tendency for a relative decrease in QoL in the intervention group when computing the index. In the absence of a value set for Switzerland, the French set was used for the index valuation, [18] which could have an impact on this result; furthermore, the population sample used to obtain this value was significantly younger (mean age of 49 years) than our population, and thus likely in a much better health state, which should also render the results hard to interpret. To clarify this effect, our study could have benefited from the addition of another instrument specially geared towards older people, as recommended by Makai and coll., [40] although this would have added to the burden of the NH staff conducing the study, which was already significant.

Similarly to QoL, the number of common complaints related to drugs tended to be higher in the intervention than in the control group at follow-up. This questionnaire was specifically devised by the investigators for use in this study and was not validated, so the reliability of this finding is low, and the difference did not reach statistical significance, but these two tendencies need to be taken into account for the design of future studies.

#### On safety

Results on the safety of the intervention are different from those found in the literature for studies that trialled similar interventions: in a 2018 meta-analysis, Kua and colleagues found that medication review-directed deprescribing interventions could reduce the risk of death by 26% in NH residents, and the number of residents experiencing at least one fall by 24% [5]. Both these results, however, come from the pooled analysis of several thousand study participants, and the much smaller sample of our study could be the cause of this difference. Another study, published too late for inclusion in the meta-analysis, that tested a similar intervention in Dutch nursing homes, with a population with very close characteristics to ours and a larger sample size (*n* = 426), also found no benefit on falls or hospitalisations [33].

### Perspectives

The clinical relevance of reducing the dose of chronic PIMs by 30% is uncertain; however, the main driver of inappropriateness in our study was the use of drugs with anticholinergic effects. According to Hilmer and coll., the cumulative dose of anticholinergic drugs received plays a role in functional decline: receiving a single anticholinergic medication for as short as two years seems to reduce gait speed and grip strength roughly equally to one supplemental co-morbidity [41]. Reducing the use of this class of drugs by 30% could thus be worthwhile, and pilot studies have shown that deprescribing interventions targeting anticholinergics could be effective and produce clinical benefits, like reducing falls and lowering depression and frailty scores [42, 43].

## Strength and limitations

Our study has a number of strengths, the main one being that the intervention was as close as possible to real-life care: although, as they collectively decided to take part in this study, the participating healthcare professionals could be considered early adopters, they did not have specific prior training relevant to the intervention besides what is commonly found in this setting in Switzerland. The pharmacists only received a 3-day education regarding the performing of medication reviews, [10] and the nurses and physicians did not receive education beyond the procedures of the study.

Another strength is the adaptability of the intervention to the local care organisation: once the deprescribing plan had been agreed on by the healthcare professionals and the resident, the details of its implementation were decided according to the local organisation of care. The variations in organisation between NHs regarding the delivery of the intervention will be further described in another paper focused on the implementation aspects of this trial.

Finally, our use of DDDs as an outcome enabled us to capture treatment changes that would not have been seen using cruder metrics like the prevalence or number of PIMs.

This trial also has some limitations; first, we did not reach the anticipated number of participants. The reluctance of representatives to allow the participation of cognitively-impaired residents was an important factor in this issue: as seen on Fig. 1, agreement to participate was more likely to be given by residents themselves (42 of the 55 approached agreed) than by their relatives (20 of 140 approached agreed). This resulted in a relatively low proportion of participants with cognitive impairment (defined as their inability to consent themselves), which is not representative of the typical NH population found in Switzerland. This small sample size limited the statistical power of our analyses, as illustrated by the large confidence intervals for some outcomes. Combined with the relatively short duration of the study, this reduced sample may also have masked some adverse influence of the intervention on safety outcomes, such as death or hospitalisation.

Some factors that may have influenced the outcomes of this study, such as the frailty or morbidity of participants, were not measured. While randomisation should ensure that all differences in participants’ characteristics between groups are the result of chance, the risk of chance confounding does increase with a smaller sample size [44]. Therefore, and while the measured characteristics of participants (see Table 1) do not indicate imbalance, we cannot exclude that some non-measured differences were present and influenced the outcomes. Given these limitations, it is therefore prudent to interpret our results as exploratory; larger studies are indeed needed to reach a definitive conclusion on the benefits and harms of deprescribing in heavily-medicated, NH-dwelling older adults.

Another potential bias results from the open-label nature of this study: the fact that the investigators, who assessed PIM status, were not blinded to allocation could have resulted in observer bias; however, as the criteria for PIMs classification were strictly defined, we evaluate this risk as low.

Finally, our study could adhere neither to the core outcome set (COS) for the trials of medication reviews in polymorbid older patients, nor to the COS for trials aimed at improving the appropriateness of polypharmacy in older people in primary care; [45, 46] this will limit the possibilities for comparing our results with further studies.

## Conclusions

This exploratory trial showed that, in heavily-medicated, NH-dwelling older adults, implementing a deprescribing plan crafted by an interprofessional clinical team following the results of a pharmacist-led, deprescribing-focused medication review, did not significantly reduce the number of PIMs used by participants. The intervention, however, showed a potential benefit in the reduction of the doses of PIMs used.

Some participants suffered adverse consequences following deprescribing, and a reduction in quality of life could not be excluded in the intervention group. Therefore, particular attention should be paid to patient’s safety and well-being in further studies of deprescribing interventions. In clinical practice, each deprescribing act should be accompanied by appropriate monitoring measures to detect these adverse consequences as soon as possible.

Anticholinergics were a large driver of inappropriateness; the impact of this intervention on their use should be further studied, as reducing their dose could lead to substantial clinical benefits.

## Supplementary information

**Additional file 1.**

## Data Availability

The datasets generated during and analysed during this trial, as well as the code used to analyse it, are available from the corresponding author upon reasonable request.

## Abbreviations

COS: Core Outcome Set
DDD: Defined Daily Dose
EQ-5D-5L: EuroQoL-5 Dimensions-5 Levels
IDeI: Individual Deprescribing Intervention
IPS: Integrated Pharmacy Service
IRR: Incidence Rate Ratio
NH: Nursing Home
PIM: Potentially Inappropriate Medication
PPI: Proton-Pump Inhibitor
QC-DeMo: Quality Circle-Deprescribing Module
QoL: Quality of Life
REDCap: Research Electronic Data Capture
START: Screening Tool to Alert to Right Treatment
STOPP: Screening Tool of Older People’s Prescriptions
