# Supplementary material for: Effects of an interprofessional deprescribing intervention in Swiss nursing homes: the Individual Deprescribing Intervention (IDeI) randomised controlled trial
Source: BMC Geriatr. 2021 Nov 19;21:655. doi: 10.1186/s12877-021-02465-7 (PMC8603597; doi:10.1186/s12877-021-02465-7)
Supplement: Supplementary file 1 — Additional file 1. [file 12877_2021_2465_MOESM1_ESM.docx]

# Supplementary table 1: STOPP and START criteria at baseline

| **Criteria** | **Overall (n=58)** | **Intervention group (n=31)** | | **Control group (n=27)** | |
| --- | --- | --- | --- | --- | --- |
|  | **n (% overall) at baseline** | **n (% in group) at baseline** | **Change at follow-up** | **n (% in group) at baseline** | **Change at follow-up** |
| **STOPP (any criteria)** | **57 (98%)** | **30 (97%)** | **-** | **27 (100%)** | **-2** |
| N1: Concomitant use of two or more drugs with antimuscarinic/anticholinergic properties | 53 (91%) | 27 (87%) | -2 | 26 (96%) | -2 |
| D5: Benzodiazepines for ≥ 4 weeks | 42 (72%) | 22 (71%) | +2 | 20 (74%) | -2 |
| K1: Benzodiazepines (sedative, may cause reduced sensorium, impair balance) | 42 (72%) | 22 (71%) | +2 | 20 (74%) | -2 |
| D8: Anticholinergics/antimuscarinics in patients with delirium or dementia | 31 (53%) | 15 (48%) | -2 | 16 (59%) | -1 |
| I1: Antimuscarinic drugs with dementia, chronic cognitive impairment, narrow-angle glaucoma, or chronic prostatism | 29 (50%) | 15 (48%) | -2 | 14 (52%) | - |
| K2: Neuroleptic drugs | 21 (36%) | 10 (32%) | - | 11 (41%) | - |
| F3: Drugs likely to cause constipation in patients with chronic constipation where non-constipating alternatives are available | 15 (26%) | 11 (35%) | - | 4 (15%) | - |
| K4: Hypnotic Z-drugs e.g. zopiclone, zolpidem, zaleplon (may cause protracted daytime sedation, ataxia). | 10 (17%) | 6 (19%) | -1 | 4 (15%) | - |
| F2: PPI for uncomplicated peptic ulcer disease or erosive peptic oesophagitis at full therapeutic dosage for > 8 weeks. | 5 (9%) | 5 (16%) | -5 | 0 (%) | - |
| J1: Sulphonylureas with a long duration of action with type 2 diabetes mellitus | 5 (9%) | 2 (6%) | -1 | 3 (11%) | - |
| L1: Use of oral or transdermal strong opioids as first line therapy for mild pain | 5 (9%) | 4 (13%) | -4 | 1 (4%) | -1 |
| B6: Loop diuretic as first-line treatment for hypertension | 4 (7%) | 3 (10%) | - | 1 (4%) | +1 |
| D14: First-generation antihistamines | 3 (5%) | 2 (6%) | -2 | 1 (4%) | -1 |
| D9: Neuroleptic antipsychotic in patients with behavioural and psychological symptoms of dementia (BPSD) unless symptoms are severe and other non-pharmacological treatments have failed | 3 (5%) | 2 (6%) | - | 1 (4%) | - |
| **START (any criteria)** | **31 (53%)** | **15 (48%)** | **-5** | **16 (59%)** | **+2** |
| E5: Vitamin D supplement in older people who are housebound or experiencing falls or with osteopenia | 9 (16%) | 5 (16%) | - | 4 (15%) | -1 |
| E4: Bone anti-resorptive or anabolic therapy (e.g. bisphosphonate, strontium ranelate, teriparatide, denosumab) in patients with documented osteoporosis, where no pharmacological or clinical status contraindication exists | 8 (14%) | 2 (6%) | - | 6 (22%) | +1 |
| E3: Vitamin D and calcium supplement in patients with known osteoporosis and/or previous fragility fracture and/or Bone Mineral Density T-scores more than -2.5 in multiple sites | 5 (9%) | - | - | 5 (19%) | +2 |
| A6: Angiotensin Converting Enzyme inhibitor with systolic heart failure and/or documented coronary artery disease | 5 (9%) | 2 (6%) | - | 3 (11%) | - |
| H1: High-potency opioids in moderate-severe pain, where paracetamol, NSAIDs or low-potency opioids are not appropriate to the pain severity or have been ineffective | 5 (9%) | 4 (13%) | -4 | 1 (4%) | -1 |
| A7: Beta-blocker with ischaemic heart disease | 4 (7%) | 2 (6%) | - | 2 (7%) | - |
| G2: 5-alpha reductase inhibitor with symptomatic prostatism, where prostatectomy is not considered necessary | 3 (5%) | 2 (6%) | +1 | 1 (4%) | - |
| A1: Vitamin K antagonists or direct thrombin inhibitors or factor Xa inhibitors in the presence of chronic atrial fibrillation | 3 (5%) | 3 (10%) | -1 | 0 (%) | +1 |
| C5: Selective serotonin reuptake inhibitor (or SNRI or pregabalin if SSRI contraindicated) for persistent severe anxiety that interferes with independent functioning | 3 (5%) | 1 (3%) | -1 | 2 (7%) | - |

Only criteria applying to 3 or more participants shown. STOPP: screening tool of older people’s prescriptions; START: screening tool to alert to right treatment; NYHA: New York Heart Association heart failure classification; SNRI: serotonin noradrenalin reuptake inhibitors; SSRI: selective serotonin reuptake inhibito
